# Mechanical Versus Bioprosthetic Conduits for the Bentall Procedure: A 10-Year Propensity Score-matched Analysis of Early and Long-Term Outcomes

**DOI:** 10.1093/icvts/ivag125

**Published:** 2026-04-22

**Authors:** Emine Aleyna Eroglu Polat, Gokay Altaylı, Mehmet Okan Donbaloglu, Mucahit Polat

**Affiliations:** Department of Cardiovascular Surgery, Mehmet Akif Ersoy Thoracic and Cardiovascular Surgery Hospital, Istanbul, 34303, Turkey; Department of Cardiovascular Surgery, Mehmet Akif Ersoy Thoracic and Cardiovascular Surgery Hospital, Istanbul, 34303, Turkey; Department of Cardiovascular Surgery, Mehmet Akif Ersoy Thoracic and Cardiovascular Surgery Hospital, Istanbul, 34303, Turkey; Department of Cardiovascular Surgery, Mehmet Akif Ersoy Thoracic and Cardiovascular Surgery Hospital, Istanbul, 34303, Turkey

**Keywords:** Bentall procedure, prosthetic valve, propensity score matching

## Abstract

**Objectives:**

The optimal conduit choice for the Bentall procedure—mechanical (MC) or biological (BC)—is confounded by significant baseline selection bias. We aimed to evaluate the impact of conduit choice on early and long-term outcomes after mitigating this bias in a contemporary surgical cohort.

**Methods:**

We retrospectively reviewed 442 consecutive patients undergoing elective Bentall procedures between 2015 and 2025 (365 MC vs 77 BC). Propensity score matching (1:1 nearest-neighbour, calliper 0.1) was performed based on 10 baseline covariates (including age, EuroSCORE II, and comorbidities) to generate a balanced cohort of 154 patients (77 per group). Operative outcomes, 30-day morbidity/mortality, long-term overall survival (OS), and reoperation-free survival (RFS) were compared using paired statistical tests and Kaplan-Meier (log-rank) analysis.

**Results:**

Propensity score matching successfully balanced baseline covariates, eliminating significant differences in age (mean 64.1 [MC] vs 65.7 [BC] years, *P* = .403) and EuroSCORE II (*P* = .698). The median follow-up was 36.0 months (interquartile range [IQR]: 18.0-78.0). In the propensity-matched cohort, no significant differences were found in long-term OS (*P* = .574) or RFS (*P* = .944) between the mechanical and biological groups. Early mortality was also comparable (*P* = .344). However, the biological group demonstrated a significantly higher rate of early re-exploration for bleeding (24.7% vs 6.5%, *P* = .003), associated with a 3.8-fold increased risk (odds ratio [OR]: 4.72, 95% confidence interval (CI), 1.66-13.40).

**Conclusions:**

In this 10-year propensity-matched analysis, both mechanical and bioprosthetic conduits demonstrated comparable long-term durability and survival outcomes. Although biological conduits were associated with a higher risk of early re-exploration for bleeding, this did not adversely affect early mortality or long-term RFS. These findings suggest that bioprosthetic conduits offer robust mid-term effectiveness, provided that meticulous intraoperative haemostasis is ensured to mitigate early bleeding risks.

**Clinical registration number:**

Not applicable (retrospective study).

## Graphical abstract

**Figure:**
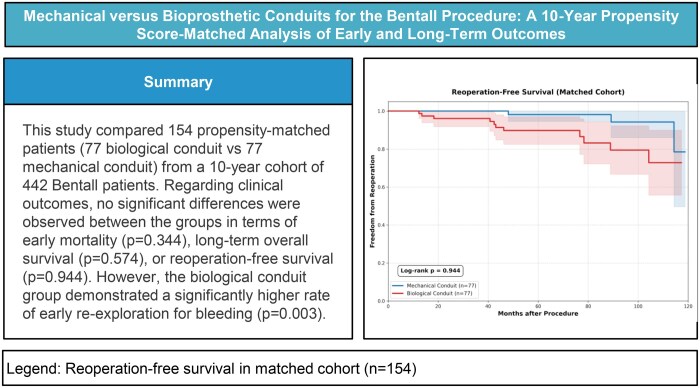

Clinical relevance statement In the ongoing debate over Bentall conduits, clinicians often assume bioprosthetics fail more while mechanical valves bleed more. Our propensity-matched study challenges this paradigm. We found no difference in long-term reoperation or survival. However, we identified a significant, unexpected 3.8-fold higher risk of early re-exploration for bleeding with bioprosthetics (*P* = .003), despite both groups being anticoagulated. This finding suggests surgeons should not be complacent about early haemostasis with biological conduits and that the long-term durability trade-off may be less pronounced than traditionally assumed in matched populations.

## INTRODUCTION

The Bentall procedure, first described by Bentall and De Bono in 1968, remains the surgical standard for concomitant replacement of the aortic valve, aortic root, and ascending aorta.^1^ Despite decades of technical refinement, the optimal choice of valved conduit remains a subject of significant clinical debate.

The selection of an optimal prosthesis, whether mechanical heart valve (MHV) or bioprosthetic heart valve (BHV), remains a critical, patient-specific decision in accordance with international guidelines. This choice requires a multifactorial assessment, balancing the patient’s age, estimated life expectancy (as determined by sex and comorbidities), lifestyle considerations, and the competing risks of thromboembolism versus major bleeding.^2^

This decision framework is dictated by the fundamental trade-off inherent to each prosthesis type. Mechanical conduits (MCs) offer superior long-term durability but mandate lifelong oral anticoagulation (OAC), which carries an inherent cumulative risk of both haemorrhagic and thromboembolic complications. Conversely, bioprosthetic conduits obviate the need for OAC but are susceptible to time-dependent structural valve degeneration (SVD), raising the lifetime probability of requiring a future reoperation.

This established paradigm has been recently modified by the advent of transcatheter therapies. The feasibility of performing a transcatheter valve-in-valve (TAVI) procedure to treat a degenerated bioprosthesis has emerged as a significant advantage for the biological option, offering a less invasive reintervention pathway for selected, often high-risk, patients.^3^

A critical challenge in comparing these 2 strategies is the inherent selection bias: patients receiving bioprosthetic conduits are typically older and present with more comorbidities. Propensity score matching (PSM) offers a robust statistical methodology to balance these baseline covariates, allowing for a more unbiased comparison.

This study aimed to assess the early and long-term operative and clinical outcomes following the Bentall procedure using either mechanical or bioprosthetic valved conduits over a 10-year period at our institution, employing PSM to minimize the impact of selection bias.

## PATIENTS AND METHODS

### Study design and patient population

This single-centre, retrospective cohort study included consecutive adult patients who underwent elective Bentall procedure at our institution between January 2015 and January 2025. A total of 848 patients were initially screened for eligibility. To ensure a homogeneous cohort of elective Bentall procedures with complete data, strict exclusion criteria were applied.

**Figure 1.**
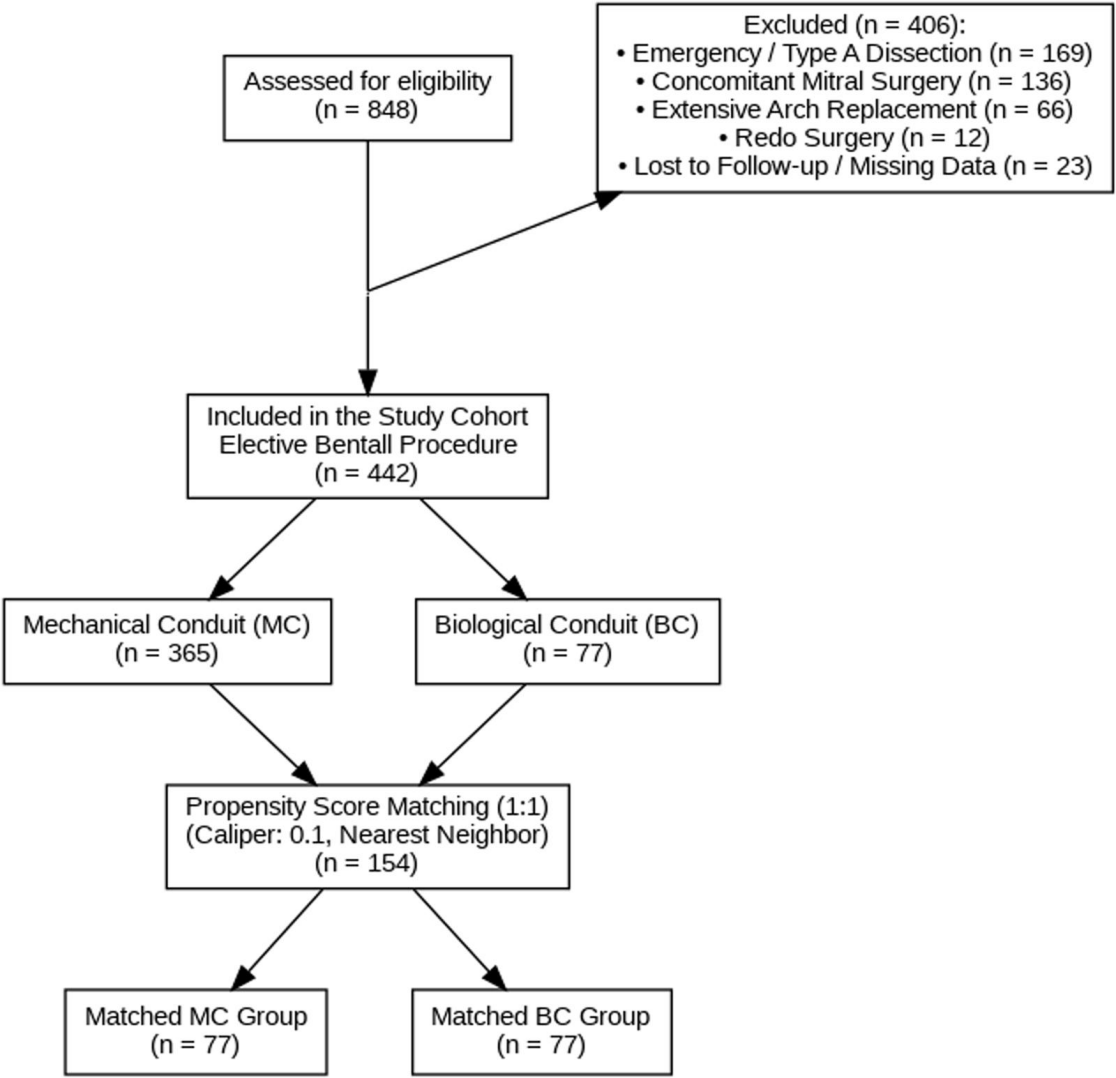
Study Flow Chart. Diagram illustrating the patient selection process, exclusion criteria, and the creation of the propensity score-matched cohorts. A total of 848 patients were screened, and 442 patients meeting the inclusion criteria for elective Bentall procedures were enrolled. Propensity score matching (1:1) resulted in 77 matched pairs. Abbreviations: BC: biological conduit; MC: mechanical conduit; PSM: propensity score matching

Patients were excluded if they presented with acute type A aortic dissection or emergency status (*n* = 169), required concomitant mitral valve surgery (*n* = 136), underwent extensive arch replacement beyond the hemiarch (*n* = 66), or were undergoing redo cardiac surgery (*n* = 12). Additionally, 23 patients were excluded due to loss to follow-up or significant missing data. The final study population consisted of 442 patients with complete data undergoing elective Bentall procedures. These patients were stratified into 2 groups based on the implanted prosthesis type: the MC group (*n* = 365) or the biological conduit (BC) group (*n* = 77) (**Figure 1**).

### Covariate and end-point definitions

Baseline patient characteristics and comorbidities were defined according to standardized criteria. Left ventricular dysfunction (LVD) was defined as a pre-operative left ventricular ejection fraction (LVEF) below 40%, as assessed by echocardiography. The diagnosis of chronic obstructive pulmonary disease (COPD) required a post-bronchodilator FEV1/FVC ratio of less than 70% on pulmonary function tests. Diabetes mellitus (DM) was defined by a pre-operative haemoglobin A1c (HbA1c) level greater than 6.0% or active use of anti-glycaemic medication. A diagnosis of Marfan syndrome was restricted only to those patients with a confirmed genetic diagnosis. Furthermore, all specific other clinical variables required for the calculation of the EuroSCORE II were systematically retrieved from individual patient records.

Early outcomes included 30-day or in-hospital mortality, re-exploration for bleeding, prolonged ventilation (>24 hours), new-onset postoperative atrial fibrillation (POAF), need for permanent pacemaker implantation, and early cerebrovascular event. Operative times (cardiopulmonary bypass [CPB] and cross-clamp) and durations of intensive care unit (ICU) and total hospital stay were recorded.

Long-term outcomes included overall survival (OS) (all-cause mortality), cardiac-related mortality, late stroke, and freedom from reoperation (defined as any resternotomy for valve-related complications). All mortality end-points were classified into 3 distinct periods based on timing relative to the index procedure, according to the Valve Academic Research Consortium 3 (VARC-3) criteria^4^: periprocedural mortality (death ≤30 days post-procedure or >30 days but during the index hospitalization), early mortality (death >30 days but ≤1 year after index hospitalization), and late mortality (death >1 year after index hospitalization).

### Ethics statement

The study was conducted in accordance with the ethical principles of the Declaration of Helsinki and the WMA Declaration of Taipei regarding ethical considerations regarding health databases and biobanks. The initial study protocol was approved by the Institutional Ethics Committee of Mehmet Akif Ersoy Thoracic and Cardiovascular Surgery Hospital (Date: May 27, 2025, IRB No: 2025.05-44). Following the finalization of the research framework, a revised approval was obtained to fully encompass the current scope and administrative requirements of the study (IRB No: 2026.04-47). Due to the retrospective nature of the study, the requirement for individual informed consent was waived by the committee.

### Surgical technique and postoperative management

All decisions regarding the indication for operation, as well as the subsequent choice of prosthesis type, were determined by a consensus decision of the multidisciplinary Heart Team. This decision was made in accordance with current international guidelines, and the final prosthesis selection was at the team’s discretion, guided primarily by patient-specific factors such as age, comorbidities, and patient preference.

Operations were performed via median sternotomy. Cardiopulmonary bypass (CPB) was typically established via distal ascending aortic and right atrial cannulation, with axillary artery cannulation utilized in selected complex cases. Systemic cooling was maintained between 28°C and 30°C. Myocardial protection was achieved using routine antegrade del Nido cardioplegia. The modified “button” Bentall technique was the standard approach.^5^ Following longitudinal aortotomy, the coronary buttons were mobilized. For the MC group, a commercially available pre-assembled valved conduit was utilized. In contrast, for the BC group, a composite graft was constructed intraoperatively by suturing the appropriate-sized stented bioprosthetic valve into a Dacron vascular graft.

The proximal anastomosis was performed using interrupted pledged 2-0 polyester (Ti-Cron) mattress sutures in a supra-annular position. Coronary buttons were re-implanted using continuous 6-0 polypropylene sutures. The distal anastomosis was completed using continuous 4-0 polypropylene sutures, reinforced with a Teflon felt strip when necessary.

Postoperative management was standardized. Patients in the MC group received lifelong warfarin (target international normalized ratio [INR]: 2.0-3.0). Patients in the BC group received warfarin for the initial 3 months postoperatively (target INR: 2.0-2.5), followed by lifelong low-dose aspirin (100 mg/day).

### Statistical analysis

Continuous variables were presented as mean ± standard deviation (SD) or median [interquartile range (IQR)] and were compared using the Student’s t-test or Mann-Whitney U test. Categorical variables were expressed as frequencies and percentages and compared using the χ^2^ test or Fisher’s exact test.

To address selection bias, a logistic regression model calculated a propensity score for each patient based on 10 pre-operative covariates: age, sex, body surface area (BSA), LVD (LVEF < 40%), NYHA class (III/IV), COPD, DM, bicuspid valve, Marfan syndrome, and EuroSCORE II. A 1:1 nearest-neighbour matching algorithm with a calliper width of 0.1 was used to create a matched cohort. Adequate balance was confirmed by achieving a standardized mean difference (SMD) < 0.1 for all covariates (except BSA, as noted).

Within the matched cohort, paired continuous variables were compared using the Wilcoxon signed-rank test, and paired categorical variables using McNemar’s test. Long-term survival (OS) and reoperation-free survival (RFS) were analysed using the Kaplan-Meier method, and curves were compared using the log-rank test. As a secondary analysis, multivariable logistic regression models were constructed using the entire unmatched cohort to identify independent predictors for key early outcomes. Effect estimates for binary outcomes are reported as odds ratios (ORs) with 95% confidence intervals (CIs). For time-to-event analyses, Hazard Ratios (HR) were estimated using Cox proportional hazards regression models.

A *P*-value < .05 was regarded as statistically significant. While our primary analyses were conducted using IBM SPSS (Version 26.0), we integrated Python (Version 3.9) into our workflow to refine the matching process and ensure high-fidelity data visualization.

## RESULTS

### Patient population and PSM

**Figure 2.**
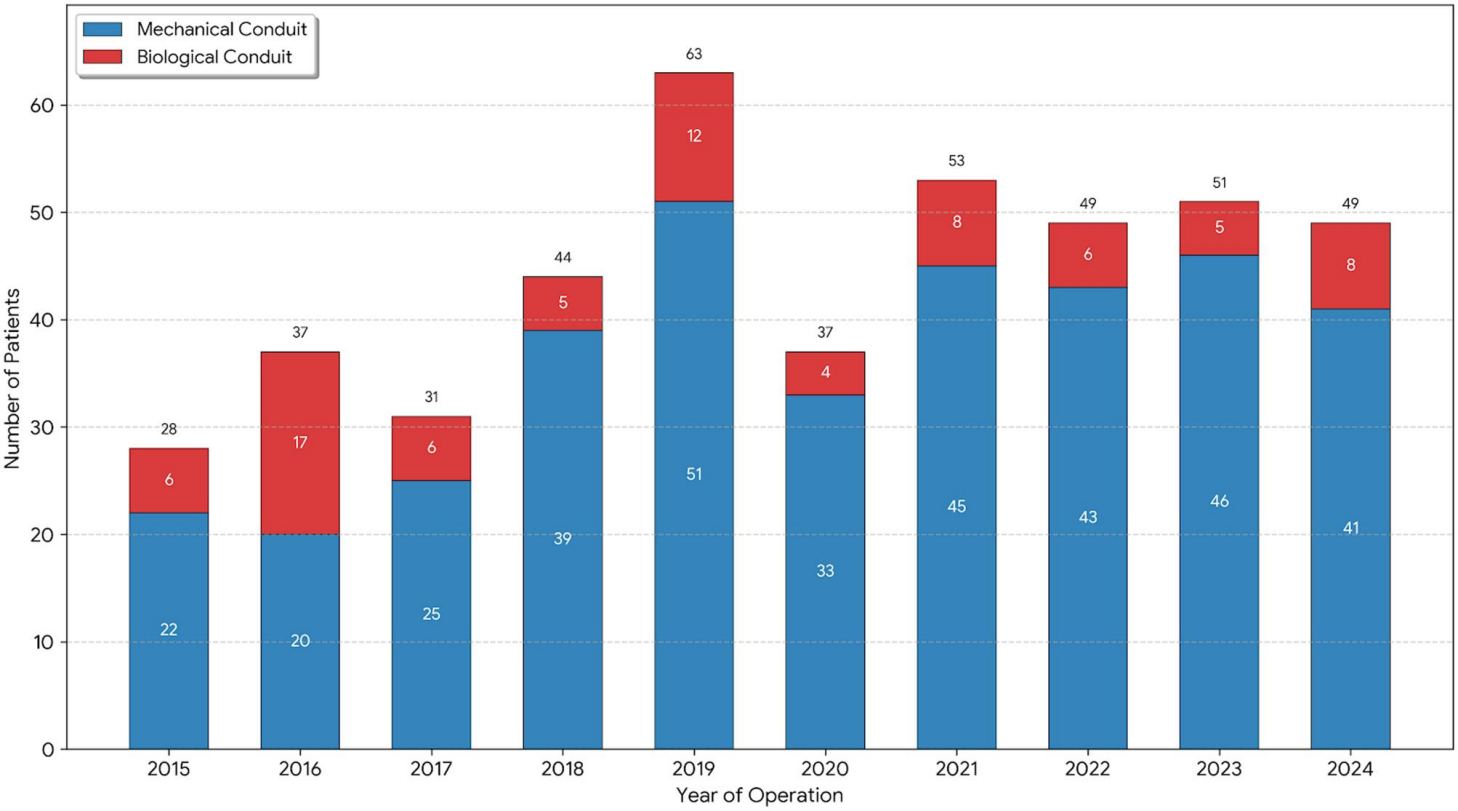
Annual Distribution of Conduit Types Over the Study Period (2015-2024). The stacked bar chart illustrates the number of patients undergoing elective Bentall procedures each year, stratified by prosthesis type

A total of 442 consecutive patients were included in the final analysis (365 MC vs 77 BC). The temporal distribution of the 442 elective Bentall procedures over the 10-year study period is presented in **Figure 2**. The implantation of BCs was observed throughout the entire study decade and was not limited to a specific short timeframe. The annual surgical volume remained relatively stable, with the exception of a transient decline in 2020, which is attributed to institutional restrictions on elective cardiac surgery during the peak of the COVID-19 pandemic.

The unmatched groups demonstrated significant baseline heterogeneity (**Table 1**). Patients in the BC group were, on average, 16 years older than those in the MC group (65.66 ± 11.45 vs 49.82 ± 12.59 years, *P* < .001) and had a significantly higher mean EuroSCORE II (2.41 ± 1.49 vs 1.76 ± 1.14, *P* < .001). After 1:1 propensity matching, a well-balanced cohort of 154 patients (77 per group) was established. (**Table S1**) As shown in **Table 1**, the PSM process successfully eliminated the significant baseline differences in age (*P* = .403), EuroSCORE II (*P* = .698), COPD (*P* = .846), DM (*P* = 1.000), and bicuspid valve status (*P* = 1.000) (**Figures S1** and **S2**).

**Table 1.** Comparison of Baseline Patient Characteristics Before and After PSM

	Mechanical (before PSM, *n* = 365)	Biological (before PSM, *n* = 77)	*P*-value	Mechanical (after PSM, *n* = 77)	Biological (after PSM, *n* = 77)	*P*-value
Age (mean ± SD)	49.82 ± 12.59	65.66 ± 11.45	<.001	64.09 ± 11.81	65.66 ± 11.45	.403
Sex (male, *n*, %)	299 (81.9%)	63 (81.8%)	1.000	54 (70.1%)	63 (81.8%)	.131
BSA (mean ± SD)	1.97 ± 0.19	1.91 ± 0.17	.029	1.85 ± 0.19	1.91 ± 0.17	.024
EuroSCORE-II (mean ± SD)	1.76 ± 1.14	2.41 ± 1.49	<.001	2.49 ± 1.67	2.39 ± 1.47	.698
COPD (*n*, %)	31 (8.5%)	18 (23.4%)	<.001	16 (20.8%)	18 (23.4%)	.846
DM (*n*, %)	46 (12.6%)	20 (26.0%)	.005	21 (27.3%)	20 (26.0%)	1.000
LVD (*n*, %)	30 (8.2%)	5 (6.5%)	.781	9 (11.7%)	5 (6.5%)	.400
NYHA (class III-IV, *n*, %)	32 (8.8%)	13 (16.9%)	.053	14 (18.2%)	13 (16.9%)	1.000
Bicuspid valve (*n*, %)	112 (30.7%)	13 (16.9%)	.021	12 (15.6%)	13 (16.9%)	1.000
Marfan syndrome (*n*, %)	9 (2.5%)	1 (1.3%)	.838	1 (1.3%)	1 (1.3%)	1.000

Abbreviations: BSA, body surface area; COPD, chronic obstructive pulmonary disease; DM, diabetes mellitus; LVD, left ventricular dysfunction; NYHA Class III-IV, New York Heart Associations Class III-IV; PSM, propensity score matching; SD, standard deviation.

### Early clinical outcomes in the matched cohort

**Figure 3.**
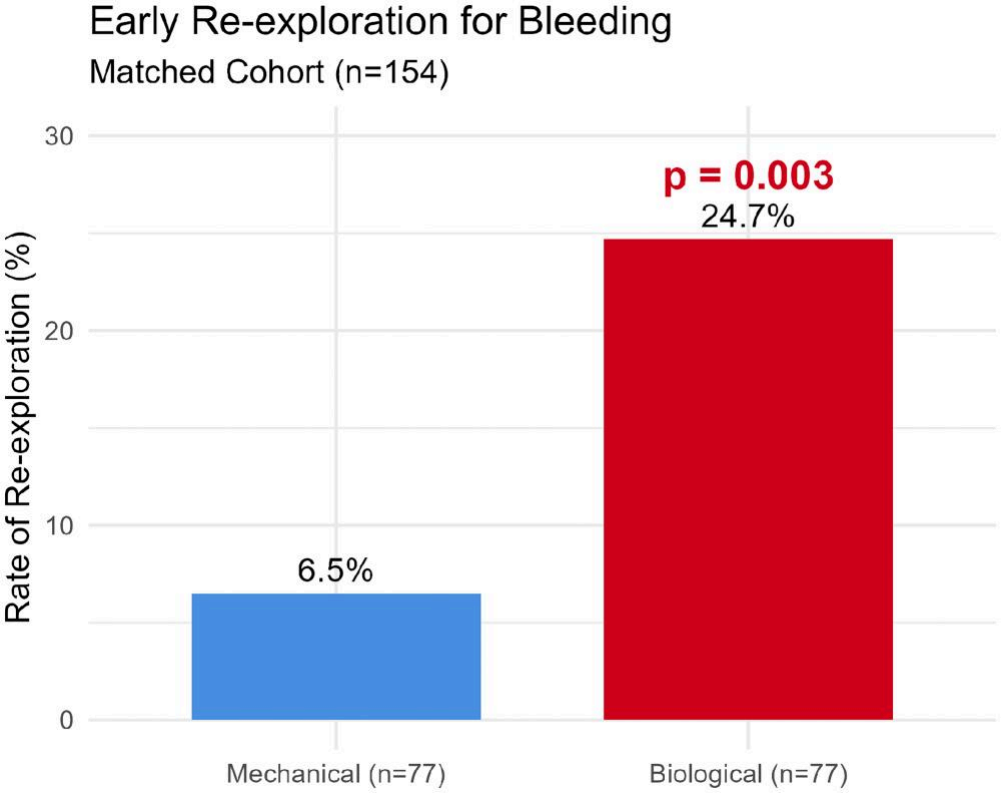
Comparison of Early Re-exploration for Bleeding Rates in the Propensity Score-matched Cohort (*n* = 154). The biological conduit group demonstrated a significantly higher rate of bleeding revision (24.7%) compared to the mechanical conduit group (6.5%). *P*-value calculated using McNemar’s test

Analysis of early clinical outcomes for the 154-patient matched cohort (**Table 2**) revealed a statistically significant difference in the rate of re-exploration for bleeding. Specifically, the biological group demonstrated a 3.8-fold increase in the rate of bleeding revision, occurring in 24.7% (*n* = 19) of patients compared to 6.5% (*n* = 5) in the mechanical group (*P* = .003; **Figure 3**). The estimated risk of re-exploration was over 4 times higher for BCs (OR: 4.72, 95% CI, 1.66-13.40).

**Table 2.** Early Clinical Outcomes in the Propensity Score-Matched Cohort (*n* = 154)

	Mechanical (*n* = 77)	Biological (*n* = 77)	*P*-value (paired)
Bleeding revision (*n*, %)	5 (6.5%)	19 (24.7%)	.003
Periprocedural mortality (*n*, %)	3 (3.9%)	7 (9.1%)	.344
Prolonged vent. time (*n*, %)	20 (26.0%)	18 (23.4%)	.851
POAF (*n*, %)	23 (29.9%)	19 (24.7%)	.711
Early stroke (*n*, %)	0 (0.0%)	1 (1.3%)	1.000
Need to PPMI (*n*, %)	1 (1.3%)	2 (2.6%)	1.000
CPB time (median, min)	154.0	141.0	.097
Cross-clamp time (median, min)	100.0	99.0	.812
ICU stay (median, days)	2.0	2.0	.922
Hospital stay (median, days)	10.0	8.0	.090

Abbreviations: CPB, cardiopulmonary bypass; ICU, intensive care unit; POAF, postoperative atrial fibrillation; PPMI, permanent pacemaker implantation.

In contrast, no statistically significant difference was observed in 30-day mortality (9.1% [BC] vs 3.9% [MC], *P* = .344). Furthermore, operative times and postoperative recovery metrics were comparable. There were no significant differences in median cross-clamp time (99.0 min [BC] vs 100.0 min [MC], *P* = .812), median CPB time (141.0 min [BC] vs 154.0 min [MC], *P* = .097), or median hospital stay (*P* = .090).

In patients requiring re-exploration for bleeding, intraoperative findings predominantly revealed diffuse mediastinal oozing without a single distinct surgical bleeding focus, particularly in the BC group.

Regarding the post-discharge period (first 3 months), a focused review of patient records revealed no occurrences of cranial bleeding in either group. However, late pericardial effusion requiring pericardiocentesis was observed in 2 patients (at 2 and 3 months postoperatively), both of whom were in the MC group. No bleeding complications requiring readmission were noted in the BC group during this specific post-discharge window.

### Long-term outcomes in the matched cohort

Follow-up was complete for all 154 matched patients. The median follow-up duration for the matched cohort was 36.0 months (IQR: 12.0-72.0 months), with a mean duration of 47.7 ± 36.1 months.

**Figure 4.**
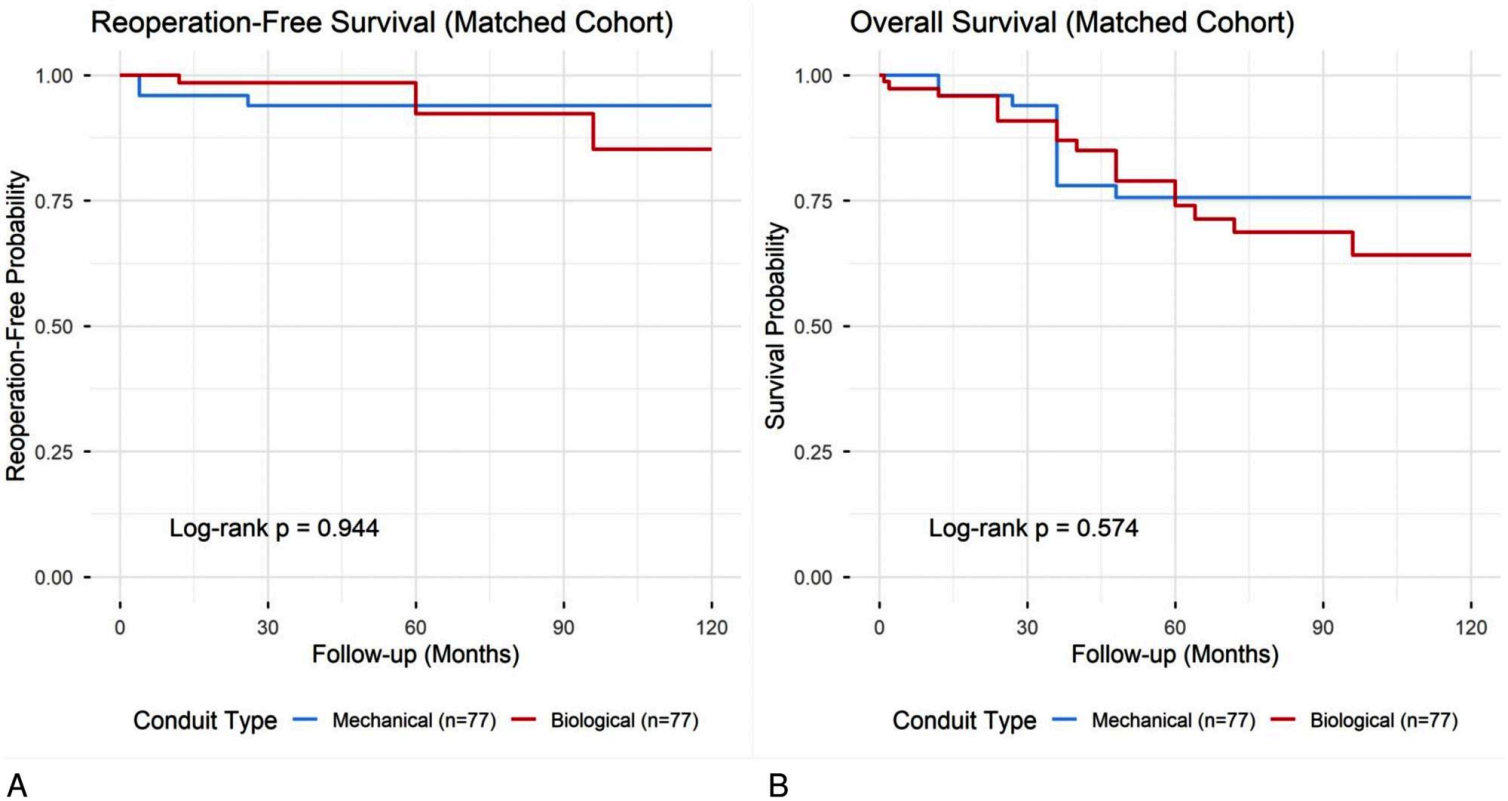
(A) Kaplan-Meier estimates freedom from reoperation in the matched cohort. Despite a higher crude number of events in the biological group, the reoperation-free survival rates were statistically comparable between the mechanical and biological groups over the follow-up period (log-rank *P* = .944). (B) Kaplan-Meier estimates of long-term overall survival in the matched cohort. There was no statistically significant difference in survival between patients receiving mechanical and biological conduits (log-rank *P* = .574)

Long-term survival and freedom from events were analysed using the Kaplan-Meier method. There was no statistically significant difference in long-term OS between the 2 groups (log-rank *P* = .574) as shown in **Figure 4B**. In the multivariable Cox regression analysis, conduit type was not found to be a significant predictor of late mortality. During the follow-up period, 11 deaths (14.3%) occurred in the biological group, compared to 18 deaths (23.4%) in the mechanical group. Similarly, Kaplan-Meier analysis revealed no statistically significant difference in RFS between the groups (log-rank *P* = .944) as shown in **Figure 4A**. A total of 11 reoperations (14.3%) occurred in the biological group, compared to 3 reoperations (3.9%) in the mechanical group.

In the mechanical group (*n* = 3), the indications for reoperation were endocarditis at 22 months, prosthesis dysfunction at 60 months, and a thoraco-abdominal aneurysm at 84 months. In the biological group (*n* = 11), the known indications included prosthesis dysfunction (*n* = 4; occurring at 10, 14, 30, and 96 months), endocarditis (*n* = 3; occurring at 14, 26, and 36 months), and one case of late tamponade due to left main coronary artery button dehiscence.

Regarding the mode of reintervention, one patient in the BC group developed severe prosthetic aortic insufficiency due to structural valve deterioration at 8 years postoperatively. This patient successfully underwent a valve-in-valve transcatheter aortic valve implantation procedure as a less invasive alternative to redo surgery.

The multivariable logistic regression model for early mortality (*n* = 442) did not identify any statistically significant (*P* < .05) independent risk factors. In contrast, the multivariable logistic regression model for re-exploration for bleeding successfully identified 2 statistically significant independent risk factors. As detailed in **Table 3**, after adjusting for all other baseline covariates, concomitant coronary artery bypass grafting (CABG) (OR 4.51, *P* < .001) and the use of a BC (OR 2.69, *P* = .023) were both independently associated with a significantly higher risk of re-exploration for bleeding.

**Table 3.** Multivariable Logistic Regression Analysis for Independent Predictors of Re-exploration for Bleeding (*n* = 440)

	Odds ratio (OR)	95% Confidence interval (CI)	*P*-value
Concomitant CABG	4.513	2.152-9.464	<.001
Biological valve	2.687	1.143-6.313	.023
Concomitant hemiarch replacement	2.525	0.898-7.098	.079
Marfan syndrome	5.188	0.741-36.341	.097
BSA	0.293	0.041-2.116	.224
Age	1.022	0.987-1.058	.225
EuroSCORE-II	0.826	0.604-1.131	.233
Sex (male)	1.891	0.648-5.519	.244
LVD	0.570	0.146-2.228	.419
Bicuspid valve	1.284	0.597-2.761	.522
NYHA Class III-IV	1.314	0.465-3.717	.606
COPD	0.804	0.296-2.187	.670
DM	1.017	0.408-2.537	.971

Abbreviations: BSA, body surface area; CABG, coronary artery bypass grafting; CI, confidence interval; COPD, chronic obstructive pulmonary disease; DM, diabetes mellitus; LVD, left ventricular dysfunction; NYHA III-IV, New York Heart Associations Class III-IV; OR, odds ratio.

## DISCUSSION

This study utilized PSM to mitigate significant selection bias in a 10-year cohort of Bentall procedures, yielding several key findings. Our study ultimately confirms that the modified Bentall procedure, when performed in high-volume centres, is a safe and durable operation associated with excellent long-term outcomes, independent of the specific conduit type selected.^6^ This conclusion is strongly supported by our primary long-term analysis, which—after rigorously balancing baseline risk factors—demonstrated no statistically significant difference in either OS (log-rank *P* = .574) or RFS (log-rank *P* = .944) between the matched cohorts.

The most striking result was the 3.8-fold increased risk of re-exploration for bleeding in the BC group (*P* = .003). At first glance, this result appears counter-intuitive, as mechanical valves are traditionally associated with higher anticoagulation-related bleeding risks.^7^ However, since our institutional protocol mandates warfarin for the first 3 months following bioprosthetic implantation, both cohorts were under similar anticoagulation regimens during the early postoperative phase. This suggests that the observed disparity in bleeding may be independent of the anticoagulation strategy itself.

While our findings contrast with several large-scale matched studies that reported comparable re-exploration rates between conduit types, we hypothesize that this increased risk is likely multifactorial.^8^ First, despite propensity matching, the biological cohort was significantly older, which may imply a higher baseline of tissue friability. Second, a key technical distinction lies in the conduit preparation; while MCs are pre-assembled, biological composite grafts are constructed intraoperatively. This adds a circumferential suture line that is inherently susceptible to needle-hole oozing.

Furthermore, the potential role of valve-mediated coagulopathy cannot be overlooked. Although all patients underwent rigorous preoperative optimization, previous literature has indicated that biological prostheses can trigger a transient postoperative reduction in platelet count.^9^ Therefore, it is plausible that a combination of subclinical postoperative thrombocytopaenia and the additional surgical suture lines contributed to the haemostatic challenges observed in the biological group.

Our second major finding was the elimination of the early mortality difference after matching. In the raw data, the biological group had a significantly higher mortality, a finding consistent with their older age and higher risk profile. After PSM, early mortality was statistically similar (9.1% vs 3.9%, *P* = .344). This finding strongly supports the primary goal of PSM: it demonstrates that the choice of a BC, when selected for an appropriately matched patient, does not inherently confer a higher early surgical risk. This result is consistent with several other propensity-matched studies of the Bentall procedure, which also reported no significant difference in early mortality between conduit types.^6^^,^^8^

Third, our long-term analysis revealed no significant difference in either OS (*P* = .574) or RFS (*P* = .944). The parity in OS is consistent with large scale, propensity-matched registry studies of the Bentall procedure, which have similarly reported comparable long-term survival regardless of the prosthesis type.^10^

The finding of equivalence in RFS is particularly noteworthy (*P* = .944). While the raw event count for reoperation was substantially higher in the biological group (11 vs 3), the Kaplan-Meier analysis, which accounts for the time-to-event, found this difference to be non-significant. This may suggest that within our median follow-up of 36.0 months, the risk of SVD-related reoperation—which typically accelerates after the first decade—did not outweigh the baseline risk of non-valve-related reoperation observed in the mechanical group.^11^

Finally, our experience confirms that BCs offer the distinct advantage of future valve-in-valve TAVI feasibility, as successfully demonstrated in one of our patients who presented with late structural valve deterioration.

## CONCLUSION

In this 10-year, propensity-matched analysis of the Bentall procedure, we sought to compare mechanical and BCs after eliminating significant baseline selection bias. Our findings challenge the traditional paradigm that the primary trade-off is mechanical valve bleeding versus biological valve failure. First, the use of a BC was associated with a distinct increase in the rate of re-exploration for bleeding during the early postoperative period. Second, this choice of prosthesis had no discernible impact on early mortality.

While survival appears comparable in the mid-term, BCs are associated with a significantly higher risk of early bleeding and a trend towards increased late reintervention. Therefore, MCs remain the gold standard for suitable patients, while biological options require careful patient selection acknowledging these trade-offs.

### Limitations

This study has several limitations. First, its retrospective, single-centre design inherently carries a risk of selection bias. Although we employed PSM, the substantial reduction in sample size represents a significant limitation. This loss of data points to a potential selection bias where only patients with comparable baseline profiles were included, inevitably reducing the generalizability of our findings to the broader, non-matched population. Furthermore, while the matching process was successful for the majority of clinical covariates, a minor residual difference in BSA remained (*P* = .024), which should be noted when interpreting haemodynamic outcomes. Third, sample size was limited in the biological group. The lack of statistical significance in long-term reoperation rates (*P* = .08) might represent a type II error due to limited statistical power rather than true equivalence.

## Supplementary Material

ivag125_Supplementary_Data

## Data Availability

The data underlying this article will be shared on reasonable request to the corresponding author.

## ABBREVIATIONS

BC: Biological conduit
BHV: Bioprosthetic heart valve
BSA: Body surface area
CABG: Coronary artery bypass grafting
CI: Confidence interval
COPD: Chronic obstructive pulmonary disease
CPB: Cardiopulmonary bypass
DM: Diabetes mellitus
HbA1c: Haemoglobin A1c
ICU: Intensive care unit
IQR: Interquartile range
INR: International normalized ratio
IRB: Institutional Review Board
LVD: Left ventricular dysfunction
LVEF: Left ventricular ejection fraction
MC: Mechanical conduit
MHV: Mechanical heart valve
NYHA III-IV: New York Heart Associations Class III-IV
OAC: Oral anticoagulation
OR: Odds ratio
OS: Overall survival
POAF: Postoperative atrial fibrillation
PSM: Propensity score matching
RFS: Reoperation-free survival
SD: Standard deviation
SMD: Standardized mean difference
SVD: Structural valve degeneration
TAVI: Transcatheter aortic valve implantation
VARC-3: Valve Academic Research Consortium 3
