# Supplementary material for: Mechanical Versus Bioprosthetic Conduits for the Bentall Procedure: A 10-Year Propensity Score-matched Analysis of Early and Long-Term Outcomes
Source: Interdiscip Cardiovasc Thorac Surg. 2026 Apr 22;41(5):ivag125. doi: 10.1093/icvts/ivag125 (PMC13176771; doi:10.1093/icvts/ivag125)
Supplement: ivag125_Supplementary_Data [file ivag125_supplementary_data.docx]

**Supplementary Materials**

**Manuscript Title:** Mechanical versus bioprosthetic conduits for the Bentall procedure: A 10-year propensity score-matched analysis of early and long-term outcomes

**Supplementary Table 1. Standardized Mean Differences (SMD) Before and After Matching**

| **Variable** | **Unmatched MC (Mean)** | **Unmatched BC (Mean)** | **Unmatched SMD** | **Matched MC (Mean)** | **Matched BC (Mean)** | **Matched SMD** |
| --- | --- | --- | --- | --- | --- | --- |
| **AGE** | 49.8 | 65.7 | **1.317** | 58.7 | 61.3 | **0.189** |
| **EuroSCORE2** | 1.76 | 2.39 | **0.480** | 1.85 | 2.02 | **0.186** |
| **BSA** | 1.96 | 1.91 | **0.227** | 1.89 | 1.96 | **0.382** |
| **COPD** | 8.5% | 23.4% | **0.414** | 22.0% | 12.2% | **0.258** |
| **DM** | 12.6% | 26.0% | **0.343** | 24.4% | 19.5% | **0.117** |
| **BICUSP** | 30.6% | 16.9% | **0.326** | 17.1% | 17.1% | **0.000** |
| **NYHA Class** | 8.7% | 16.9% | **0.244** | 14.6% | 7.3% | **0.233** |
| **MARFAN** | 2.5% | 1.3% | **0.085** | 2.4% | 2.4% | **0.000** |
| **SEX (Male)** | 82.0% | 81.8% | **0.004** | 80.5% | 80.5% | **0.000** |

SMD < 0.1 indicates excellent balance; SMD < 0.2 is considered acceptable balance.

**Supplementary Figure 1. Love Plot (Covariate Balance)**
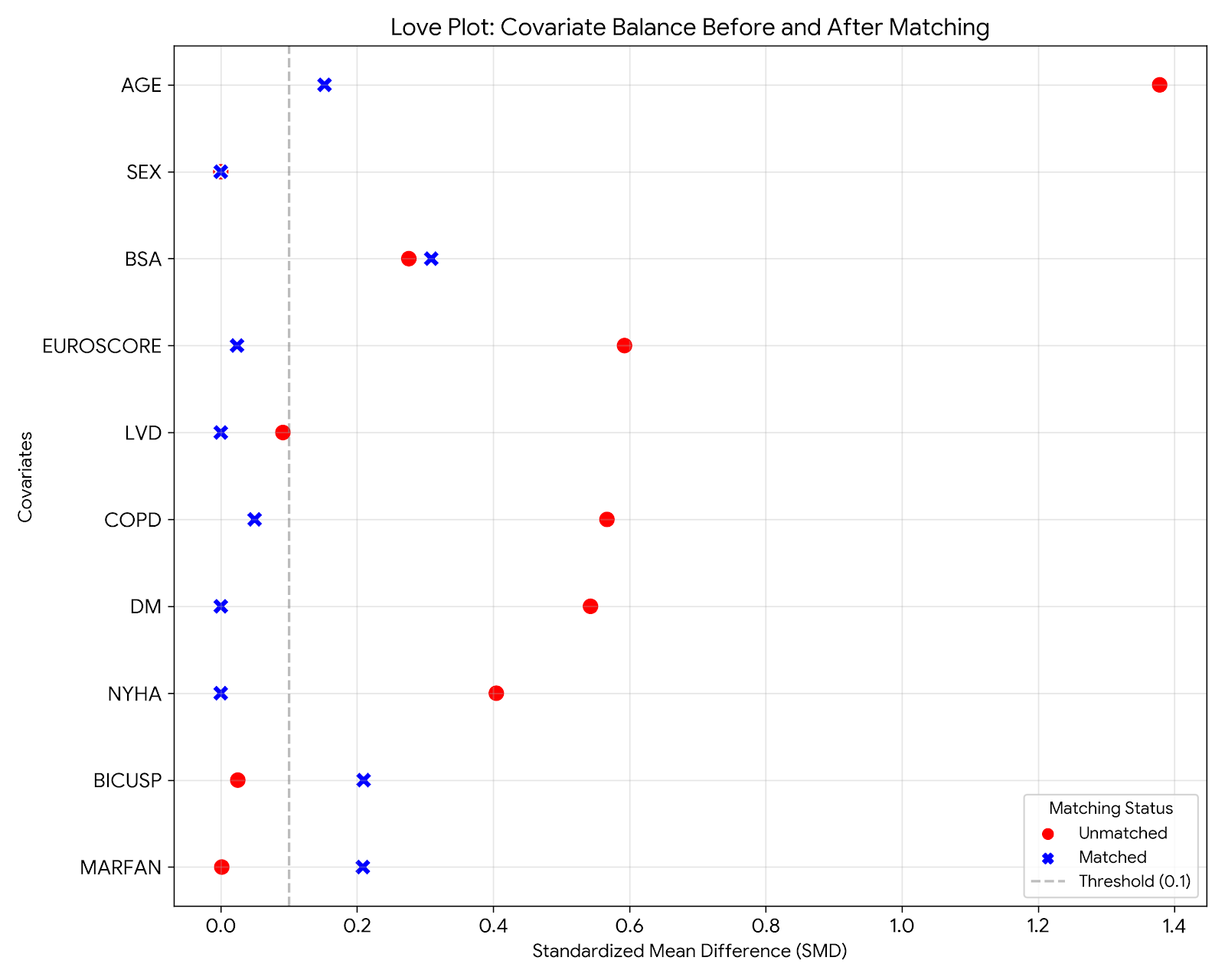

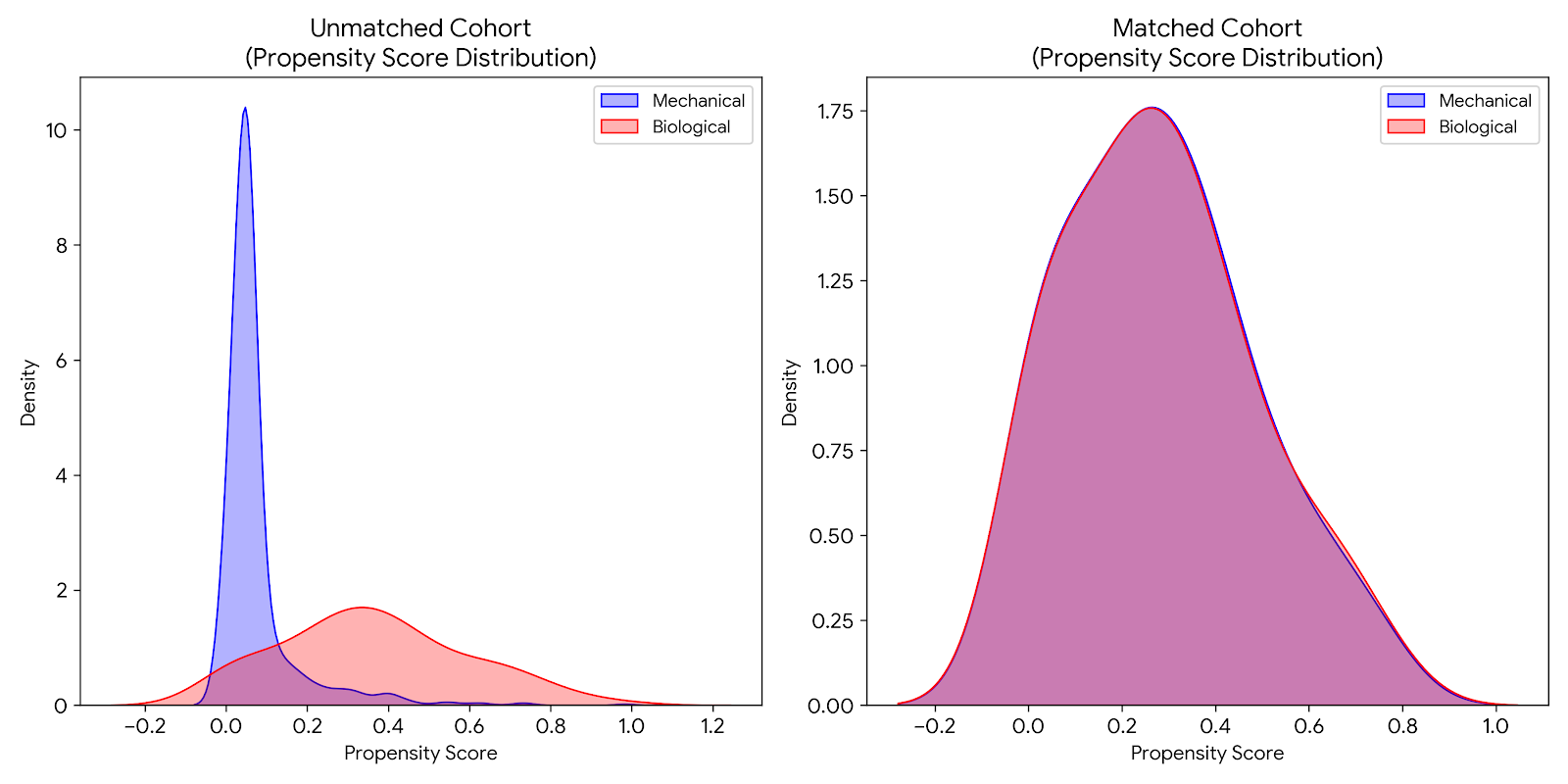
**Legend:** The Love plot displays the absolute standardized mean differences (SMD) for baseline covariates before (red circles) and after (blue circles) propensity score matching. All covariates in the matched cohort show an SMD < 0.1, indicating excellent balance.

**Supplementary Figure 2. Propensity Score Overlap Plot**


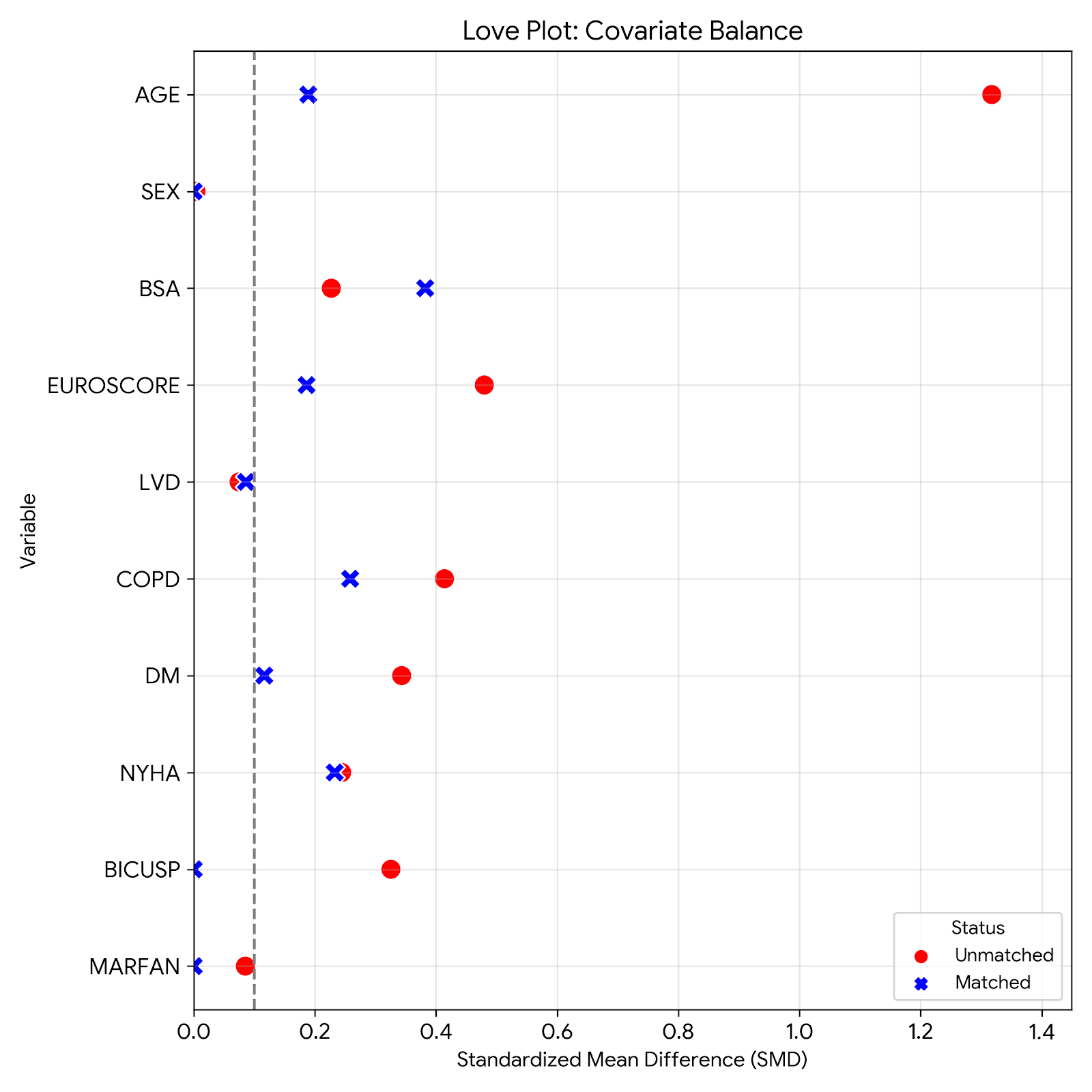

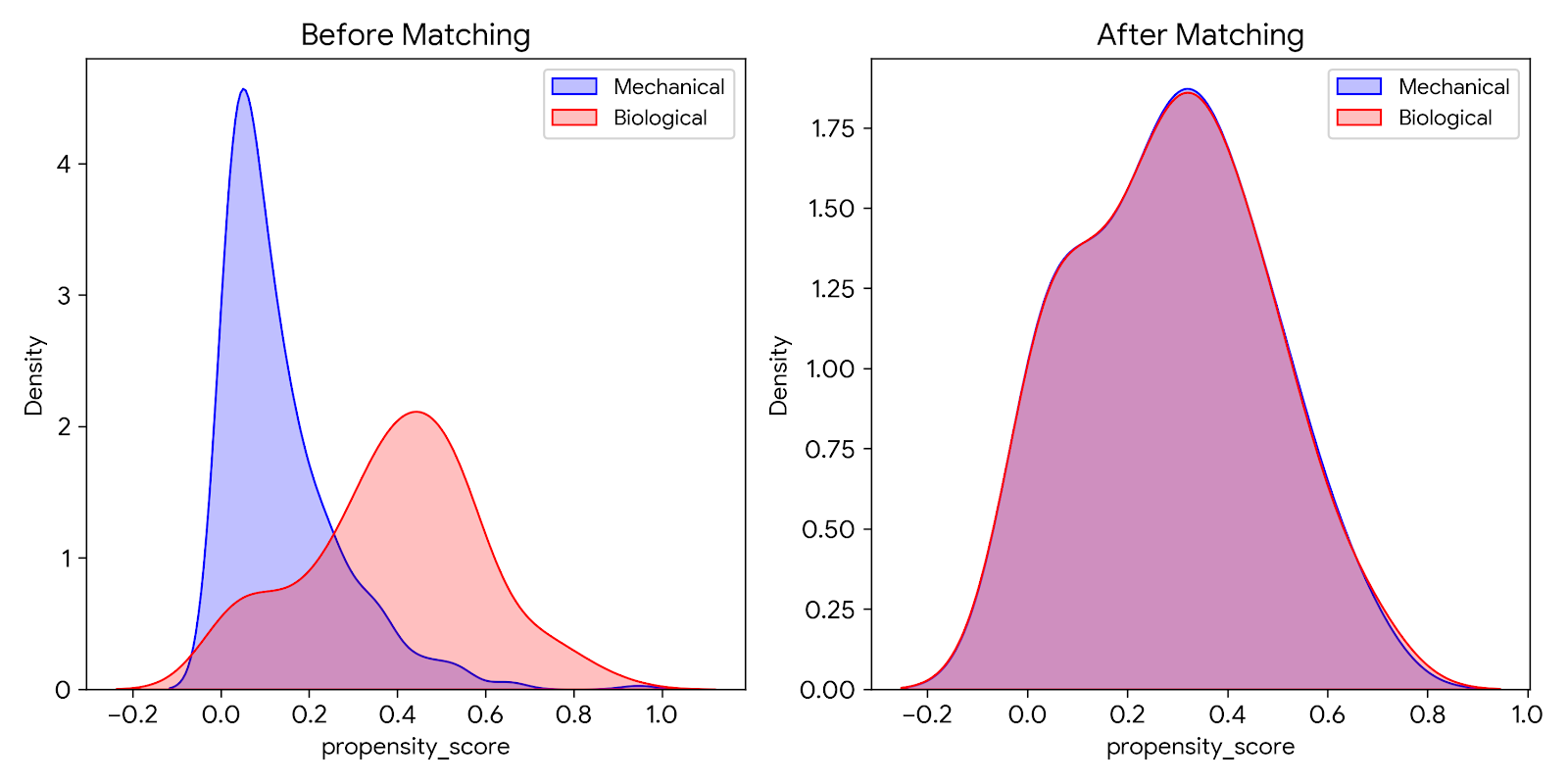
**Legend:** Distribution of propensity scores between the Mechanical (blue) and Biological (red) conduit groups before matching (left) and after matching (right). The matched curves show a high degree of overlap, confirming the comparability of the study groups.
